# Aging Regulates Receptivity by Modulating the Expression of Osteopontin and HOXA10 in the Human Endometrium

**DOI:** 10.3390/jcm15093402

**Published:** 2026-04-29

**Authors:** Fanourios Makrygiannakis, Maria Marmara, Thomas Vrekoussis, Dragana Nikitovic, Antonios Makrigiannakis, Aikaterini Berdiaki

**Affiliations:** 1 Department of Histology-Embryology, Division of Morphology, School of Medicine, University of Crete, 70013 Heraklion, Greece; fanouris007@gmail.com (F.M.); marmaramaria25@gmail.com (M.M.); nikitovic@uoc.gr (D.N.); 2 Laboratory of Human Reproduction, Department of Obstetrics and Gynecology, University of Crete, 71003 Heraklion, Greece; vrekoussis@gmail.com (T.V.); makrygia@uoc.gr (A.M.)

**Keywords:** primary stromal cells, endometrium, osteopontin, CD44, HOXA10, age, receptivity

## Abstract

**Background/Objectives**: Aging is increasingly recognized as a key determinant of changes in human tissue and cellular function. Women’s age, in particular, has been associated with reduced oocyte quality and negatively correlated with the expression of genes involved in endometrial decidualization and cellular function. The ability of endometrial cells to interact and allow the invasion of the growing embryo is defined as endometrial receptivity. Investigating age-related differences in human endometrial receptivity may expand our understanding of factors contributing to infertility. **Methods**: Stromal cells were isolated and cultured from endometrial pipelle biopsies (*n* = 28) obtained from female donors at the proliferative phase of the menstrual cycle. Protein and mRNA expression of the receptivity modulators OPN, CD44, and HOXA10 were analyzed by Western blot and real-time PCR, respectively. **Results**: Data presented a linear decrease in mRNA expression of OPN and HOXA10 (*p* = 0.0066, R^2^ = 0.3018 and *p* = 0.0036, R^2^ = 0.529, respectively) with women’s increasing age, and a similar trend was evident at the protein level (OPN, *p* < 0.05; HOXA10, *p* < 0.01). Further analysis of the data included separating the samples into three age groups: 25–35 years, 36–40 years, and 41–46 years. ANOVA revealed a significant decrease in OPN and HOXA10 mRNA expression (*p* = 0.03158 and *p* = 0.02578, respectively). CD44 expression did not differ with age. **Conclusions**: OPN and HOXA10 are negatively correlated with increasing maternal age. These findings suggest that age-related alterations in key endometrial receptivity modulators may contribute to impaired implantation and could represent potential targets for diagnostic or therapeutic strategies in human implantation failure.

## 1. Introduction

Age-related infertility is a worldwide phenomenon in modern reproductive medicine [1,2]. Increasing scientific data highlight age-associated declines in fertility, including reduced conception rates and lower live birth outcomes from autologous oocytes in women of advanced reproductive age undergoing in vitro fertilization [3,4,5,6,7,8]. In almost half of the infertility cases, the causes are attributed to the embryo’s genetic material and possible immune cell reactions. Accordingly, the remaining proportion is attributed to anatomical, uterine, cervical, lifestyle, and environmental factors [9,10]. There is increasing evidence on the role of the inner uterine layer, the endometrium, in the age-related increase in female infertility, and this study aims to extrapolate its precise contribution. The cyclic changes of the endometrium during the menstrual cycle include the initial proliferation of stromal cells and their subsequent differentiation/decidualization, which is under the influence of the ovarian hormonal control [11]. During the menstrual cycle, there is differential activation and interplay among several signaling molecules that lead to an optimal receptive endometrium for embryo implantation [12]. Implantation rates have been negatively correlated with age, in studies using euploid embryos [13] and, more recently, mathematical models [14]. Jiang et al. also reported a decline in live birth rates with increasing maternal age following single euploid embryo transfer [15]. Studies specifically investigating the expression of age-related factors in endometrial stromal cells are limited and have examined molecules involved in decidualization, receptivity, proliferation, cytoskeletal formation, and cell survival [16,17,18,19]. Endometrial receptivity describes the capacity of the endometrium to accept the invading blastocyst/embryo [20]. Factors that modulate this ability are involved in embryo-endometrial interactions, such as osteopontin (OPN), an extracellular matrix glycoprotein, and the transmembrane receptor CD44. Variation in OPN and CD44 expression during the menstrual cycle has been described in mice and humans [21,22,23], but studies concerning the molecular pathways involved are scarce. CD44 has been investigated in animal models, showing its importance in embryonic initial attachment [24]. OPN expression in the mouse uterus was proven crucial for blastocyst attachment to the endometrium, and further inhibition of OPN in endometrial stromal cells led to impaired trophoblast invasion [25]. Furthermore, OPN has been shown to play a key role in uNK killer cell function in the uterus during decidualization [26]. More importantly, OPN-CD44 adhesion complex’s levels were modulated in ovulatory human PCOS patients, affecting endometrial infertility [27]. The same study reported crosstalk between the CD44-OPN adhesion complex, ERα, STAT1, and NF-κB pathways that regulated endometrial receptivity [27]. Beyond their individual roles, increasing evidence supports a functional interplay between OPN and CD44, forming a ligand–receptor axis that integrates extracellular matrix signaling with intracellular pathways regulating endometrial function [28,29]. OPN binds directly to CD44 isoforms at the cell surface, promoting the formation of adhesion complexes that modulate cytoskeletal organization, cell adhesion, and migration [28,30]. This interaction has been implicated in the spatial organization of signaling platforms, facilitating receptor clustering and activation of downstream pathways, including MAPK, NF-κB, and STAT signaling cascades [29,30]. In the endometrium, such mechanisms are particularly relevant, as dynamic remodeling of the extracellular matrix and coordinated stromal–epithelial communication are essential for establishing a receptive state [31]. Moreover, both OPN and CD44 are regulated by steroid hormones, positioning this axis at the interface between hormonal control and local microenvironmental cues [32]. Dysregulation of the OPN–CD44 interaction has been associated with impaired implantation processes, altered immune cell function, and pathological conditions such as PCOS, further supporting its role as a key mediator of endometrial receptivity [27] (Paravati et al., 2020). Collectively, these findings highlight the OPN–CD44 axis as a novel, central molecular regulator of endometrial receptivity, orchestrating embryo attachment, trophoblast invasion, immune cell function, and signaling crosstalk critical for successful implantation.

Additional regulators include cytokines, growth factors, hormone signaling pathways, cell adhesion molecules, and transcription factors such as homeobox A10 (HOXA10) [33,34]. HOXA10 plays a critical role in the development of the female reproductive tract [35]. In Hoxa10 knockout mice, severe defects in decidualization result in female infertility [36], accompanied by impaired steroid receptor function that compromises implantation [37]. Furthermore, HOXA10 is regulated by progesterone in human endometrial stromal cells [38] and has been shown to control stromal cell proliferation in mice [39].

Difficulties in implantation and low pregnancy rates have been linked to diminished endometrial receptivity associated with advancing maternal age [40,41,42,43]. Previous studies have reported a downregulation of the receptivity gene HOXA10 and decidualization factors STAT3 and BMP2 with increasing uterine age [16,17]. In addition, studies on endometrial cellular senescence of stromal cells have correlated aging with reduced endometrial receptivity [44].

Because endometrial receptivity is critical for successful embryo implantation, identifying how it changes with uterine aging will provide a strong basis for understanding pregnancy complications associated with advancing age. However, the molecular mechanisms underlying age-related alterations in stromal cell function remain incompletely understood. To elucidate the role of endometrial cell aging in endometrial receptivity, the present study investigated age-associated changes in the mRNA and protein expression of OPN, CD44, and HOXA10 in primary human endometrial stromal cells in vitro.

## 2. Materials and Methods

### 2.1. Materials

Enzymes used for tissue digestion, Collagenase (C9891) and DNase I (11284932001), were purchased from Sigma-Aldrich (St. Gallen, Switzerland). Primary antibodies from Santa Cruz Biotechnology (Heidelberg, Germany) used were anti-OPN (mouse monoclonal; 1/100 dilution; sc-21742), anti-CD44 (mouse monoclonal; 1/100 dilution; sc-9960), and anti-HOXA10 (mouse monoclonal; 1/100 dilution; sc-271428). In addition, anti-actin (MAB1501; mouse monoclonal; 1/2500 dilution) and secondary-HRP anti-mouse antibody (AP192PM; 1/4000 dilution) by Millipore (Darmstadt, Germany) were utilized. Opti-Protein marker (abm, Vancouver, BC, Canada; G252) was used as a protein marker.

### 2.2. Human Tissues

Pipelle endometrial tissue biopsies (*n* = 28) (Table 1) were collected from ovulating fertile (at least one child) European female donors, aged 25–46 years old (mean age 39 y). All the endometrial biopsies were collected during the proliferative phase (days 7–9) of the menstrual cycle. Variables between the two age groups for Table 1 were tested using a Student’s *t*-test. Inclusion criteria for all women were regular menstrual cycle, normal uterine cavity, normal endometrial thickness on ultrasound, and normal levels (according to age) of serum FSH, LH, oestradiol, progesterone, anti-Müllerian hormone (AMH), and prolactin. The exclusion criteria were uterine abnormalities, hormone or metabolic disorders, and autoimmune diseases. Also, women were not prescribed any hormonal treatment for at least 3 months before the Pipelle biopsy was carried out. Informed written consent was obtained from each patient.

Initially, the samples were collected from women aged 25 to 46 years, and the aim was to correlate the expression of receptivity factors with increasing age. The next analysis of the data included separating samples into three age groups based on the key age thresholds of 35 and 40 years, which are associated with decreased female infertility. Thus, three age groups used for mRNA expression were 25–35 y, 36–40 y, and 41–46 y.

Variables between samples for Table 1 were tested and found homogenous: for BMI, *p* = 0.2257; for menstrual cycle day, *p* = 0.2481; and for menstrual cycle duration, *p* = 0.8252. Therefore, the differences observed in this study were solely due to age variation. The study was conducted in accordance with the Declaration of Helsinki, and the use of human tissue was approved by the Bioethics Committee of the University General Hospital of Heraklion (PAGNI) (09/20-03-2019, 12285 28/04/2023, and 1298 08/01/2024).

### 2.3. Isolation of Primary Endometrial Stromal Cells

Endometrial tissue biopsies were collected into 5 mL of complete DMEM/F-12 (Gibco, Grand Island, NY, USA; 31330-038) supplemented with 10% FBS (charcoal stripped; Biosera, Cholet, France; FB-1001F), 1% antibiotics/antimycotics (Gibco; 15240-062), 1% L-glutamine (Gibco; 25030024), 1 nM β-estradiol (Sigma-Aldrich, (St. Gallen, Switzerland); Ε2758) and 2 μg/mL recombinant human insulin (Sigma-Aldrich; 91077C). The isolation protocol was performed according to Berdiaki et al., 2022 [16]. The enzymes used were 0.5 mg/mL collagenase (Sigma-Aldrich; C9891) and 0.1 mg/mL DNase I (Roche, Basel, Switzerland; 11284932001) for 1 h at 37 °C (5% CO_2_). Isolated cells were filtered through a 40 µm cell-strainer (Falcon, Corning, New York, NY, USA), which separated the epithelial cells from the primary stromal cells, red blood cells and immune cells. The resulting pellet was placed into one Τ75/T25 tissue culture flask and cultured at 37 °C and 5% CO_2_. By changing the culture media after 6–18 h, blood cells, non-adherent immune cells and tissue debris were removed [45].

### 2.4. RNA Isolation and Real-Time PCR

Total RNA isolation was performed using TRIzol (15596026; Invitrogen, Carlsbad, CA, USA), according to the manufacturer’s instructions. One microgram of RNA was added for cDNA synthesis using the TAKARA (Kusatsu, Shiga, Japan; RR037A) RT cDNA synthesis kit. For semi-quantification of the genes of interest, real-time PCR was performed on an Mx300P cycler using the Universal qPCR kit (KK4602; KAPABiosystems, Cape Town, South Africa) and specific primers (Table 2) for each gene analyzed. PCR conditions for amplification used: 94 °C for 15 min; 40 cycles at 94 °C for 20 s; 55 °C for 30 s; 72 °C for 30 s and 72 °C for 10 min. Standard curves were generated for each optimized assay, yielding a linear plot of threshold cycle (Ct) versus log(dilution). The amount of each target was quantified using the standard curve and presented as arbitrary units. GAPDH was utilized as a housekeeping control gene [46,47,48].

### 2.5. Western Blot Analysis

Primary stromal cells (PSCs) from a T25 culture flask (1st passage) were lysed using RIPA solution (50 mM Tris-HCl, 1% NP-40, 0.25% Na-Deoxycholate, 150 mM NaCl, 1 mM EDTA with protease and phosphatase inhibitors). Equal amounts of proteins (20 μg), measured using Bradford reagent (B6916; MERK, Darmstadt, Germany) at 595 nm absorbance, were subjected to SDS-PAGE using 8% polyacrylamide gels under reducing conditions (16 V for 2 h). Separated protein bands were transferred to nitrocellulose membranes in 10 mM CAPS, containing 10% methanol. Membranes were blocked overnight at 4 °C with 5% low-fat milk powder in PBS-0.1%–Tween. All first antibodies were incubated for 1 h at room temperature in 1% milk-PBS-0.1% Tween. The immune complexes were detected after incubation with the appropriate peroxidase-conjugated secondary HRP antibody (anti-mouse, AP192PM, 1/4000 dilution; Millipore, Darmstadt, Germany) and using the LumiSensor Chemiluminescent HRP substrate kit (GenScript, Rijswijk, Holand; L00221V500), according to the manufacturer’s instructions. Protein expression of β-ACTIN was used to correct for the amount of each sample analyzed. ImageJ (Version 1.52v) was used to analyze images captured by a ChemiDoc XRS+ BIO-RAD (Hercules, CA, USA) molecular imager.

### 2.6. Statistical Analysis

All experiments were repeated at least 3 times for each patient or treatment used in the in vitro culture studies. For comparisons between sample age groups, a one-way ANOVA was used with Tukey’s multiple comparisons test (to test between all age groups). The GraphPad InStat software (GraphPad 9.0; San Diego, CA, USA) was used for analysis. *p* values < 0.05 were considered significant. For the linear regression analysis R^2^ values, 0.00–0.09 were considered very weak, 0.10–0.29 weak, 0.30–0.49 moderate, and 0.50–1.0 strong effect.

## 3. Results

### 3.1. Expression Levels of Osteopontin in Relation to Women’s Age

**Figure 1:**
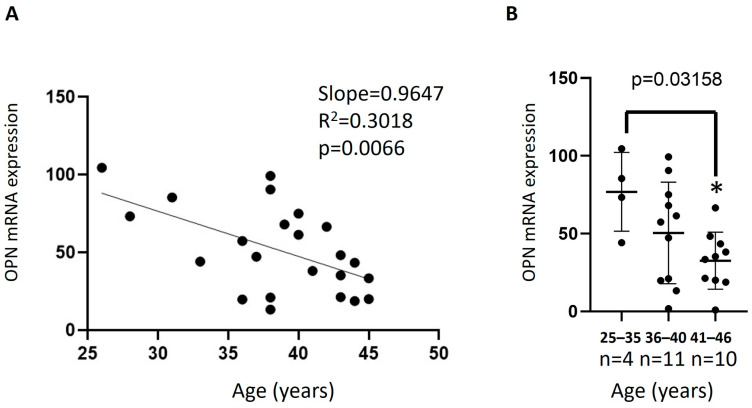
Effect of age on the expression of OPN. OPN mRNA levels in PSCs were determined by Real-Time PCR with OPN-specific primers and normalized to GAPDH. (**A**) linear correlation of samples OPN expression with increasing age; (**B**) OPN mRNA expression of the age groups (25–35 y, 36–40 y, and 41–46 y). Means ± S.E.M. were plotted to determine statistical significance between groups: * = *p* ≤ 0.05 compared with the respective age group.

In order to examine the possible correlation between increasing women’s age and mRNA expression of the OPN gene, Real-Time PCR was performed using specific primers and cDNAs from 28 collected biopsies of PSCs. The data showed a statistically significant decrease (*p* = 0.0066) in OPN primary stromal cell expression levels with increasing age, with a moderate linear correlation (R^2^ = 0.3018) (Figure 1A).

When data were divided into three age groups (25–35 y, 36–40 y, and 41–46 y), a statistically significant decrease (*p* = 0.03158) in OPN PSC expression levels with increasing age (Figure 1B). Women in the 41–45-year age group showed a significant downregulation of OPN expression compared to the younger 25–35-year age group (*p* ≤ 0.05; Figure 1).

### 3.2. Expression Levels of CD44 in Relation to Women’s Age

**Figure 2:**
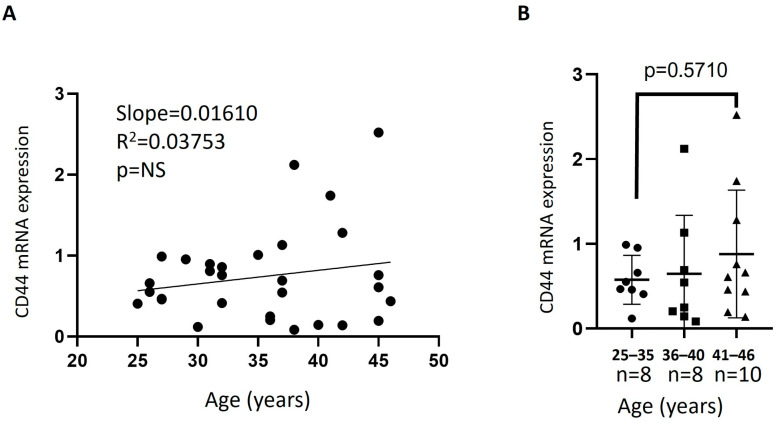
Effect of age on the expression of CD44. CD44 mRNA levels in PSC were determined by Real-Time PCR using primers specific to the CD44 gene and normalized against GAPDH. (**A**) linear correlation of samples CD44 expression with increasing age; (**B**) CD44 mRNA expression of the age groups (25–35 y, 36–40 y, and 41–46 y). Means ± S.E.M were plotted; NS = no significance.

Results for mRNA expression of CD44 in the isolated PSC samples (Table 1) and across the three age groups (25–35 y, 36–40 y, and 41–46 y) showed no significant change with age (Figure 2).

### 3.3. Expression Levels of HOΧA10 in Relation to Women’s Age

**Figure 3:**
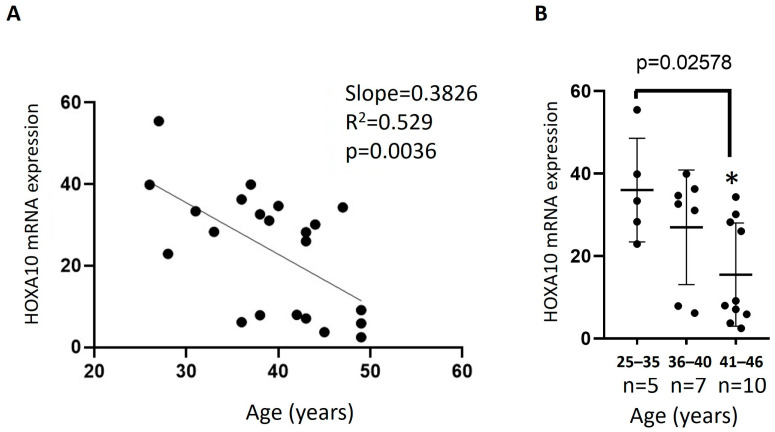
Effect of age on the expression of HOXA10. HOXA10 mRNA levels in PSCs were determined by Real-Time PCR using primers specific to HOXA10 and normalized to GAPDH. (**A**) linear correlation of samples HOXA10 expression with increasing age; (**B**) HOXA10 mRNA expression of the age groups (25–35 y, 36–40 y, and 41–46 y). Means ± S.E.M. were plotted to determine statistical significance between groups: * = *p* ≤ 0.05 compared with the respective age group.

The third factor examined was HOXA10. mRNA expression data showed a statistically significant decrease (*p* = 0.0036) in HOXA10 expression levels in primary stromal cells with increasing age, with a strong linear correlation (R^2^ = 0.529) (Figure 3A). When the data were divided into three age groups (25–35 y, 36–40 y, and 41–46 y), a statistically significant decrease (*p* = 0.02578) in HOXA10 PSC expression levels with increasing age was observed (Figure 3B). Women in the 41–45-year age group showed significant HOXA10 downregulation compared to the younger age group (25–35 years; *p* ≤ 0.05) (Figure 3B).

### 3.4. Changes in Protein Expression Levels of OPN, CD44 and HOXA10, with Increasing Age

**Figure 4:**
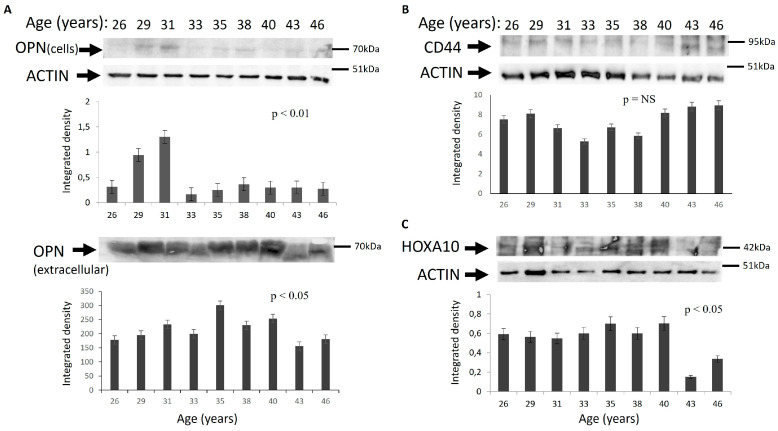
Protein expression of (**A**) OPN, (**B**) CD44 and (**C**) HOXA10 compared to women’s age groups. Protein samples of isolated PSCs lysed using RIPA solution were analyzed using Western blot analysis. Densitometric analysis of OPN (cells and extracellular; MW: 25–70 kDa), CD44 (90–95 kDa) and HOXA10 (42 kDa), protein bands were normalized against ACTIN (44 kDa) and plotted. Means ± S.E.M were plotted; *p* values between all samples calculated by one-way ANOVA analysis.

Protein expression of the receptivity molecules was also assessed using a using sample of increasing age. Western blot analysis of OPN, CD44 and HOXA10 protein was performed and analyzed (Figure 4). In the case of OPN protein expression, the secreted protein was also analyzed by concentrating the supernatant from stromal cell cultures. Expression of OPN in cells decreased from the age of 33 years, while the secreted protein gradually decreased from the age of 38 years (Figure 4). CD44 protein expression, similarly to the mRNA expression, did not show differences with age. HOXA10 protein expression decreased, particularly after age 41.

## 4. Discussion

The current study showed a decrease in the expression of OPN and HOXA10, two receptivity modulators, with increasing female age in primary human endometrial cells. PSCs were isolated at the proliferative phase of the menstrual cycle from biopsies of fertile donors.

Factors expressed at the proliferative stage aid the proliferation of the functional layer of the endometrium and the endometrium’s transition to the differentiation phase, the secretory phase. Furthermore, aging is a complex biological process that leads to a decline in the function of most human organs [43,49,50,51]. These age-associated changes may reflect alterations in hormonal responsiveness, epigenetic regulation of gene expression, and progressive remodeling of the endometrial extracellular matrix, all of which are known to impact stromal cell function and decidualization capacity.

In this study, OPN, CD44 and HOXA10 were selected to be examined based on their proven [52] role during the proliferative as well as the secretory phase of the endometrial cycle. Changes in endometrial stromal cell proliferation and in the expression of decidualization factors with women’s age have been reported in cells isolated during the proliferative menstrual phase [16].

In this study’s results, women’s age was associated with receptivity modulators. Primary endometrial stromal cells were isolated and cultured from fertile women at the proliferative phase of the menstrual cycle. mRNA expressions of OPN and HOXA10 decreased with increasing age. Nevertheless, CD44 which interacts with OPN during implantation showed no age-related difference in expression levels. This finding suggests that ligand availability (OPN), rather than receptor expression (CD44), may represent the limiting factor for effective implantation signaling in the aging endometrium.

Regarding OPN protein levels, there was an in vitro decrease with increasing women’s age in primary human endometrial stromal cells. The intracellular OPN began to decline from the age of 33 years, whereas the secreted form of this protein, which plays a key role in embryo implantation, showed a reduction from the age of 38 years. This discrepancy suggests that the intracellular OPN reservoir diminishes earlier, whereas the decrease in extracellular OPN may occur later due to mechanisms that remain to be elucidated. As a matricellular protein, OPN operates within the extracellular matrix network, and its age-dependent decline may reflect broader alterations in extracellular matrix composition and signaling, which are increasingly recognized as key regulators of endometrial receptivity. This further supports the concept that age-related alterations in the endometrial microenvironment, rather than isolated gene expression changes, contribute to impaired receptivity.

The interaction of OPN with integrin αvβ3 is recognized as a critical determinant of endometrial receptivity, particularly in conditions such as repeated implantation failure (RIF) and polycystic ovary syndrome (PCOS) [15,53,54,55,56,57,58].

HOΧA10 belongs to the homeobox (HOX) gene family, which modulates embryonic development and differentiation through its coding proteins [33,59,60]. HOXA10, specifically, is known to have a crucial role in the proliferation and differentiation of endometrial cells in the human body and is considered a receptivity marker [52,61]. In vivo experiments in mouse models and in vitro studies in human cell lines establish that reduced HOXA10 levels affect endometrial epithelial cell proliferation, stromal cell decidualization failure and altered gene expression (reviewed in [33]). Studies also showed decreased expression of HOΧA10 with age [17,19]. Woods et al. (2017) reported the decrease in HOXA10 expression in mice [19]. Fogle et al. (2010) showed a decrease in HOXA10 in human stromal cells with age when the cells were treated with hCG in vitro, and the majority of the 14 women tested were between the ages of 43 and 49 (mean age was 44; small age variation) [17]. In addition, women received cyclical estrogen and progesterone before undergoing the endometrial biopsy on day 7 of progesterone administration [17]. Our data confirmed a decrease in HOXA10 mRNA expression in a larger sample size, including individuals aged 26–43 years. Furthermore, PSC protein expression experiments confirm the decrease in HOXA10 mRNA expression and show a reduction in PSC protein levels after the age of 43.

It must be noted that numerous additional factors modulate endometrial stromal cell function and differentiation, including immune cells and cytokines, extracellular matrix proteins, transcription factors, and adhesion molecules [52]. Therefore, the investigation of these factors and their age-associated alterations would further clarify the contribution of maternal age to endometrial function. Additionally, future studies should include endometrial stromal cells isolated from the secretory phase of the menstrual cycle, during which maximal receptivity is achieved within the window of implantation.

Other limitations of the study include the low sample size, the exclusive use of proliferative-phase stromal cells, and the in vitro experimental conditions. Furthermore, functional assays—such as adhesion assays between endometrial stromal cells (derived from biopsies across different age groups) and trophoblast cells, as well as proliferation and invasion assays following modulation of OPN and HOXA10 expression—are required to substantiate the role of these molecules in endometrial function, directly. In conclusion, the present findings support the concept that advanced maternal age could affect specific embryo receptivity markers and, therefore, receptivity and successful embryo implantation. While endometrial receptivity testing is presented in recent studies as a necessary tool to increase pregnancy, implantation, and live birth rates [62], the present study does not assess clinical endpoints such as implantation or live birth rates; thus, the translational implications of these findings require further validation. Our data suggest that treatment strategies could be implemented during the proliferative phase of the cycle to target endometrial receptivity factors, thereby promoting successful endometrial decidualization. Within this context, HOXA10 serves as a central molecular bridge between hormonal cues and endometrial receptivity, as it is a direct target of progesterone and can also be regulated by granulocyte-macrophage colony-stimulating factor (GM-CSF) [63,64,65]. Furthermore, the presence of regulatory elements for estrogen receptor α (Erα) in the promoter regions of both OPN and CD44 suggests that these genes are directly modulated by estrogen and progesterone, with progesterone receptor isoform B (PRB) acting specifically on OPN [66]. Overall, these findings position OPN, CD44, and HOXA10 as hormone-responsive regulators of endometrial receptivity; however, their potential as biomarkers or therapeutic targets should be considered preliminary and requires validation in studies incorporating clinical outcomes such as implantation success and live birth rates. Accordingly, future studies integrating molecular findings with clinical endpoints will be essential to determine their relevance in improving fertility, particularly in the context of advanced maternal age.

## Figures and Tables

**Table 1:** Information on female donors used in mRNA expression analysis.

Variable	Women (Mean ± SD)
Number of samples of Pipelle endometrial biopsy (volunteers)	28
Age (years)	25–46 ± 1.02
BMI * (kg/m^2^)	20.9–24.2 ± 0.64
Menstrual cycle day (days)	7–9 ± 0.13
Menstrual cycle duration (days)	25–30 ± 0.35

* Body mass index.

**Table 2:** Real-time PCR primers and amplification conditions.

Primers	Sequences
GAPDH	Forward: 5′-GGAAGGTGAAGGTCGGAGTCA-3′ Reverse: 5′-GTCATTGATGGCAACAATATCCACT-3′
OPN	Forward: 5′-GAGGGCTTGGTTGTCAGC-3′ Reverse: 5′-CAATTCTCATGGTAGTGAGTTTTCC-3′
CD44	Forward: 5′-GGTCCTATAAGGACACCCCAAAT-3′ Reverse: 5′-AATCAAAGCCAAGGCCAAGA-3′
HOXA10	Forward: 5′-GGTTTGTTCTGACTTTTTGTTTCT-3′ Reverse: 5′-TGACACTTAGGACAATATCTATCTCTA-3′

## Data Availability

The raw data supporting the conclusions of this article will be made available by the authors on request.

## Abbreviations

The following abbreviations are used in this manuscript:

AMH	Anti-Müllerian hormone
BMI	Body mass index
CD44	Cluster of Differentiation 44
FSH	Follicle stimulating hormone
GAPDH	Glyceraldehyde-3-phosphate dehydrogenase
HOXA10	Homeobox A10
HRP	Horseradish Peroxidase
LH	Luteinizing hormone
OPN	Osteopontin
PSCs	Primary stromal cells
